# Plasma β_2_-microglobulin and cerebrospinal fluid biomarkers of Alzheimer’s disease pathology in cognitively intact older adults: the CABLE study

**DOI:** 10.1186/s13195-023-01217-6

**Published:** 2023-04-01

**Authors:** Yi-Ming Huang, Ya-Hui Ma, Pei-Yang Gao, Zhi-Bo Wang, Liang-Yu Huang, Jia-Hui Hou, Lan Tan, Jin-Tai Yu

**Affiliations:** 1 Department of Neurology, Qingdao Municipal Hospital, Qingdao University, Qingdao, China; 2 National Center for Neurological Diseases in China, Department of Neurology and Institute of Neurology, Huashan Hospital, Shanghai Medical College, Fudan University, 12Th Wulumuqi Zhong Road, Shanghai, 200040 China

**Keywords:** Alzheimer’s disease, β_2_-Microglobulin, Biomarkers, Neurodegeneration

## Abstract

**Background:**

Previous studies have suggested a correlation between elevated levels of β_2_-microglobulin (B2M) and cognitive impairment. However, the existing evidence is insufficient to establish a conclusive relationship. This study aims to analyze the link of plasma B2M to cerebrospinal fluid (CSF) Alzheimer’s disease (AD) biomarkers and cognition.

**Methods:**

To track the dynamics of plasma B2M in preclinical AD, 846 cognitively healthy individuals in the Chinese Alzheimer’s Biomarker and LifestylE (CABLE) cohort were divided into four groups (suspected non-AD pathology [SNAP], 2, 1, 0) according to the NIA-AA criteria. Multiple linear regression models were employed to examine the plasma B2M’s relationship with cognitive and CSF AD biomarkers. Causal mediation analysis was conducted through 10,000 bootstrapped iterations to explore the mediating effect of AD pathology on cognition.

**Results:**

We found that the levels of plasma B2M were increased in stages 1 (*P* = 0.0007) and 2 (*P* < 0.0001), in contrast to stage 0. In total participants, higher levels of B2M were associated with worse cognitive performance (*P* = 0.006 for MMSE; *P* = 0.012 for MoCA). Moreover, a higher level of B2M was associated with decreases in Aβ_1–42_ (*P* < 0.001) and Aβ_1–42_/Aβ_1–40_ (*P* = 0.015) as well as increases in T-tau/Aβ_1–42_ (*P* < 0.001) and P-tau/Aβ_1–42_ (*P* < 0.001). The subgroup analysis found B2M correlated with Aβ_1–42_ in non-*APOE ε4* individuals (*P* < 0.001) but not in *APOE ε4* carriers. Additionally, the link between B2M and cognition was partially mediated by Aβ pathology (percentage: 8.6 to 19.3%), whereas tau pathology did not mediate this effect.

**Conclusions:**

This study demonstrated the association of plasma B2M with CSF AD biomarkers as well as a possible important role of Aβ pathology in the association between B2M and cognitive impairment, particularly in cognitively normal individuals. The results indicated that B2M could be a potential biomarker for preclinical AD and might have varied functions throughout various stages of preclinical AD progression.

**Supplementary Information:**

The online version contains supplementary material available at 10.1186/s13195-023-01217-6.

## Introduction

Alzheimer’s disease (AD) is a prevalent form of dementia characterized by a long pre-clinical stage, multiple etiologies, and pathological progression. Despite extensive research, the complex nature of this neurodegenerative disorder has made it challenging to develop effective interventions to prevent or mitigate the pathological changes associated with AD [1, 2]. This devastating illness causes significant harm to both individuals affected by the clinical symptoms and their caretakers and imposes a significant economic burden on society [3]. As such, identifying risk factors for cognitive decline in AD is crucial for preventing the progression of this disease.

Recently, it has become widely recognized that minimally invasive blood biomarkers for screening in the preclinical stage are crucial for future Alzheimer’s disease (AD) treatment [4–7]. In order to achieve early diagnosis of AD, the search for suitable blood biomarkers should be actively pursued. Previous studies have proposed β_2_-microglobulin (B2M), a subunit of primary histocompatibility complex class I (MHC I) molecules, regulates behavior, synaptic plasticity, and brain development [8–11]. The results from animal studies have emphasized that the local or systemic injection of exogenous B2M in young rats leads to neurodevelopmental and hippocampus-dependent cognitive impairments and that an increase in B2M levels was observed in aged mice, which was verified by cerebrospinal fluid (CSF) or plasma from healthy populations [12, 13]. Therefore, the possibility of plasma B2M as a blood biomarker of AD deserves to be investigated. In recent studies, Dominici et al. determined that plasma B2M levels were higher in AD patients compared to the healthy control group [14]. Similar results were found in men, with B2M levels increasing with age and being higher in the dementia group compared to the healthy group [15]. Additionally, an increase in B2M was detected in the cerebrospinal fluid of HIV-related dementia patients [16, 17]. Although these results provide some evidence of a potential relationship between B2M and cognitive function and AD, the connection remains unclear. To date, there has been no systematic cohort study explaining the relationship between B2M and cognitive dysfunction and CSF AD biomarkers.

Therefore, our study used 846 cognitively normal individuals from the Chinese Alzheimer’s Biomarker and LifestylE (CABLE) study to (1) explore the relationship between B2M levels and cognition, (2) delve into the relationship between B2M levels and CSF AD biomarkers, and (3) investigate whether the effects of B2M levels on cognitive function are mediated through AD core pathology.

## Methods

### The CABLE study

Participants in this research who demonstrated cognitively normal were given data from the CABLE study. The project, which was started in 2017, aims to find the genetic and environmental factors that influence AD biomarkers in Han Chinese, laying the groundwork for early disease detection and illness prevention. Each patient underwent a comprehensive clinical, psychiatric-psychological, psychosocial, and psychiatric assessment and the collection of biological samples (blood and CSF samples). The CABLE database research design was authorized by the Qingdao Municipal Hospital Institutional Review Board in compliance with the Helsinki Declaration. Every subject gave their informed permission.

### Participants

Participants in CABLE were Han Chinese between the ages of 40 and 90. The following conditions precluded participation: (1) central nervous system infections, multiple sclerosis, head trauma, neurodegenerative diseases other than AD (e.g., Parkinson’s, epilepsy), or other major neurological disorders; (2) significant psychiatric disorders; (3) severe systemic disease (e.g., malignancy); and (4) a history of genetic disorders in the family. The Mini-Mental State Examination (MMSE) and the Montreal Cognitive Assessment (MoCA) were used to conduct an initial cognitive screening of the subjects. Neuropsychological tests together with thorough results from CSF biomarkers and magnetic resonance imaging (MRI) were used by medical professionals with standardized training to examine all diagnoses of cognitive impairment.

There were 1844 CABLE individuals with normal cognitive functions whose covariate data were available (age, sex, years of education, and *APOE* genotypes). Due to a lack of B2M data, 625 people were initially disqualified, and 373 participants without CSF biomarkers and those whose data fell outside of the standard deviation (SD) of four times were also disqualified. Eventually, this cross-sectional study comprised 846 individuals.

### Plasma B2M determination

B2M was determined by a latex-enhanced immunoturbidimetric assay using an automated biochemical analyzer (Beckman Coulter Automated Biochemistry Analyzer AU5800, USA) following a 12-h overnight fast, using customary regular clinical laboratory techniques at the clinical chemistry department’s lab at Qingdao Municipal Hospital in China.

### CSF AD biomarker assessments

After an overnight fast, CSF specimens were collected from participants by lumbar puncture. After 2 h of collection, these samples were centrifuged at 2000* g* for 10 min to remove cells and other insoluble materials, and they were then immediately frozen at − 80 °C until testing. The thaw/freeze cycle was not allowed to go longer than twice. Within 6 months, the time from sample collection to measurement was under control. CSF amyloid β plaques 42 (Aβ_1–42_), phosphorylated tau protein (P-tau), and total tau protein (T-tau) levels were measured by the ELISA kits (Innotest β-AMYLOID (1-42) [catalog number: 81583]; β-AMYLOID (1-40) [catalog number: 81585]; PHOSPHO-TAU (181p) [catalog number: 81581]; hTAU-Ag [catalog number: 81579]; Fujirebio, Ghent, Belgium). All measures were carried out by trained laboratory staff unaware of the clinical data. Each panel also contains blank controls, run validation controls, and internal control samples in addition to the cerebrospinal fluid samples. The plate will be reanalyzed if a quality control sample is insufficient. The mean of the duplicate analyses of standards and cerebrospinal fluid samples was utilized for further statistical analyses. The intra-batch coefficient of variation (CV) was less than 5%; the mean CVs for Aβ_1–42_, P-tau, and T-tau were 4.4%, 3.5%, and 4.7%, respectively. The mean inter-batch CVs for Aβ_1–42_, P-tau, and T-tau were less than 20%, 13.4%, and 14.2%, respectively. The levels of these CSF biomarkers were not related to the sampling collection time, storage period, and intra- and inter-batch CV, according to quality control assessments.

### Evidence of AT group

The 2011 National Institute on Aging-Association Alzheimer’s (NIA-AA) committee reached the conclusion that the diagnostic criteria for preclinical AD include normal cognition but aberrant AD biomarkers [18]. Around one-third of older persons with cognitively normal brains exhibit AD pathology, according to previous amyloid imaging [11, 19, 20] and neuropathology [21, 22] investigations. Studies of Asian people have shown a similar range [23–25]. The three biomarkers in the procedure are neurodegeneration (T-tau), aggregated AD-tau (P-tau), and aggregated Aβ (Aβ_1–42_). Also, each biomarker is classified as either normal or pathological using binarization. Therefore, the cutoff values for abnormal CSF AD biomarkers were defined as Aβ_1–42_ < 205.82 pg/ml (lower third), P-tau > 48.73 pg/ml (upper third), and T-tau > 221.84 pg/ml (upper third) for T-tau. In order to limit the number of groups, the aggregated tau and neurodegeneration groups were merged, resulting in four distinct combinations of biomarker groups, including stage 0, stage 1, stage 2, and suspected non-AD pathology (SNAP) [26, 27]. Stage 0 individuals had normal Aβ_1–42_, P-tau, and T-tau readings. Stage 1 individuals had an aberrant Aβ_1–42_ but normal P-tau or T-tau. Stage 2 individuals had an abnormal Aβ_1–42_ and clear P-tau or T-tau abnormalities. Those with neurodegeneration or tau aggregation but normal amyloid levels were classified as SNAP.

### *APOE ε4* genotyping assessment

The QIAamp® DNA Blood Mini Kit was used to extract DNA from blood samples after a fast. The DNA was then divided and stored at − 80 °C in an enzyme-free EP tube until the study’s *APOE* genotyping was completed. The restriction fragment length polymorphism technique was used for genotyping two particular loci associated with *APOE ε4* status (rs7412 and rs429358). Participants were split into two groups based on their *APOE ε4* status (whether or not they had at least one copy of the *APOE ε4* gene).

### Statistical analysis

We utilized the Box-Cox transformation [28] to convert B2M into a normal distribution since it did not suit the normal distribution (Shapiro–Wilk test in R, *P* < 0.001). Additional file 1: Fig. S1 illustrates the *Q*-*Q* plot. We carried out the analysis after eliminating the outliers, which were identified as values that were 4 SD above or below the averages, in order to reduce the influence of extreme values.

In order to demonstrate the baseline characteristics of the recruited individuals, we computed and presented the number and percentage for the categorical variables, as well as the mean and standard deviation (SD) for the continuous variables. First, the difference between the AT groups of stages 0, 1, and 2 and SNAP was investigated using the chi-square analysis for categorical data, as well as the one-way ANOVA analysis and non-parametric testing for continuous variables [29, 30]. Then, intergroup differences in age (categorized as 65 years old, mid-life; 65 years old, late-life), sex (categorized as female and male), and *APOE ε4* status (categorized as 0, 1, and 2) were studied using the GraphPad Prism software, where Student’s *t*-test was used to test for comparisons between the two groups. Meanwhile, for multiple comparisons of three or more groups, one-way ANOVA and Tukey Honest significant post hoc analyses of variance were used on an age-adjusted basis. After adjusting for age, sex, educational level, and *APOE ε4* carrier status, a linear regression model was used to investigate the connection between B2M and CSF AD biomarkers and cognition (adjustment for MMSE was incorporated into the relationship between B2M and CSF AD biomarkers).

To evaluate whether AD pathology [31] mediated the relationship of B2M with cognition, we fitted linear regression models according to Baron and Kenny’s methodologies [32]. Regarding the three equations, (1) the CSF AD biomarkers (the mediator) were regressed on the B2M (independent variable or IV), (2) the cognitive score (dependent variable or DV) was regressed on the IV, and (3) the DV was regressed on the mediator and IV variables. The four-part criteria below must be simultaneously met to establish the mediation effects: (a) diminished relationship between B2M and cognition when incorporating the mediator (CSF AD biomarkers) into the regression model, (b) the significant correlation of CSF AD biomarkers to cognitive measures, (c) the significant correlation of B2M to cognitive measures, and (d) the significant correlation of B2M to CSF AD biomarkers. Subsequently, we estimated the indirect effects or attenuation, determining the significance by utilizing 10,000 bootstrapped iterations. For this model, each path controlled for *APOE ε4* status, education, sex, and age. Interaction analysis was applied to assess the influences of age, sex, year of education, cardiovascular factors (CVF), subjective cognitive decline (SCD), and *APOE ε4* status on the above relationships.

The traditional and significant two-sided *P*-value criterion was reported as 0.05. All the aforementioned statistical techniques and the creation of the diagrams were performed using the R Studio software (version 4.2.1), GraphPad Prism (version 9.4.2), Stata (version 15.1), and SPSS (version 26.0.0.0).

## Results

### Essential characteristics and intergroup comparisons

A total of 846 participants from the CABLE study were assessed, specifically for stages 0 (846), 1 (189), and 2 (92) and SNAP (250). Table 1 details the clinical characteristics and demographic features. Regarding the findings reported in the literature, the outcomes in the hypothetical group, specifically the resulting proportions of individuals, were found to be comparable [27]. With regard to the study cohort, 42% were female, aged between 40 and 90 years (mean SD = 63.1 ± 10.4), who had 9.2 ± 4.2 years of schooling and a 15.5% *APOE ε4*-positive rate. Moreover, the research population had an MMSE score of 27.7 ± 2.3 and a MoCA score of 22.7 ± 2.3 in terms of cognitive function. In reference to sex and *APOE ε4* status, no differences were found between the two groups. Stage 2, however, had older participants and featured worse cognitive composite scores than stage 0.

**Table 1:** Characteristics of participants by biomarker framework

Characteristics	Stage 0	Stage 1	Stage 2	SNAP	*P*
*N*	315	189	92	250	< 0.001
Age (years)	61.89 ± 10.78	61.39 ± 10.753	68.48 ± 9.44	66.22 ± 9.75	< 0.001
Female gender (*N*, %)	127 (40.3)	94 (49.7)	37 (48.0)	87 (37.6)	0.088
Education (years)	9.18 ± 4.11	9.61 ± 4.01	7.73 ± 4.60	8.7 ± 4.53	0.010
*APOE ε4* carriers (*N*, %)	33 (10.4)	23 (12.1)	17 (18.4)	37 (14.8)	0.193
MMSE score	27.2 ± 3.69	27.46 ± 3.06	25.46 ± 4.19	26.77 ± 3.46	< 0.001
MOCA	22.83 ± 5.21	22.81 ± 4.80	19.78 ± 5.83	21.71 ± 5.36	< 0.001
CVF (yes/*N*)	148/167	94/95	51/41	135/115	0.294
Nephropathy (yes/*N*)	7/308	2/187	4/88	7/180	0.361
Hyperlipidemia (yes/*N*)	13/302	5/184	4/88	21/229	0.033
CHD (yes/*N*)	44/271	27/162	15/77	41/209	0.829
Stroke (yes/*N*)	17/298	8/181	3/89	17/232	0.508
Diabetes (yes/*N*)	44/271	35/154	12/80	51/199	0.125
Hypertension (yes/*N*)	114/201	77/112	41/51	111/139	0.177
Smoking status (yes/*N*)	85/230	41/148	20/72	74/176	0.216
Alcohol habit (yes/*N*)	97/218	44/145	21/71	70/180	0.217
Blood biomarkers					
BUN (nmol/l)	5.74 ± 1.91	5.69 ± 2.06	5.87 ± 1.81	5.83 ± 2.35	0.510
Cr (μmol/l)	65.70 ± 25.51	66.71 ± 37.86	66.00 ± 22.95	68.17 ± 49.72	0.348
BUN/Cr	0.09 ± 0.02	0.09 ± 0.42	0.09 ± 0.03	0.09 ± 0.23	0.972
UA (μmol/l)	345.94 ± 93.97	337.98 ± 99.02	333.77 ± 82.65	347.74 ± 86.86	0.261
β_2_-microglobulin (mg/l)	1.77 ± 0.77	2.11 ± 1.89	2.36 ± 1.26	2.13 ± 2.43	< 0.001
CSF biomarkers					
Aβ_1–42_ (pg/ml)	341.24 ± 121.61	131.23 ± 44.51	135.54 ± 43.53	423.95 ± 176.26	< 0.001
Aβ_1–40_ (pg/ml)	6203.42 ± 3745.72	4882.97 ± 2774.09	7766.22 ± 4393.87	8790.70 ± 3670.17	< 0.001
T-tau (pg/ml)	156.91 ± 36.05	135.39 ± 37.95	311.79 ± 146.80	291.72 ± 101.41	< 0.001
P-tau (pg/ml)	37.33 ± 7.45	33.04 ± 8.46	59.47 ± 15.18	58.00 ± 15.04	< 0.001
Aβ_1–42_/Aβ_1–40_	0.057 (0.044–0.077)	0.030 (0.020–0.042)	0.019 (0.012–0.030)	0.048 (0.037–0.064)	< 0.001
T-tau/Aβ_1–42_	0.483 (0.371–0.631)	1.002 (0.749–1.483)	2.160 (1.521–3.356)	0.680 (0.488–0.973)	< 0.001
P-tau/Aβ_1–42_	0.116 (0.089–0.152)	0.249 (0.193–0.359)	0.441 (0.324–0.583)	0.143 (0.106–0.190)	< 0.001

Categorical variables are reported as numbers and percentages; continuous variables are reported as means ± SDs

*Abbreviations*: *MMSE *Mini-Mental State Examination, *MOCA *Montreal Cognitive Assessment, *APOE* *apolipoprotein E, CHD *coronary heart disease, *CVF *cardiovascular factors (including stroke, coronary heart disease, hypertension, diabetes), *BUN *blood urea nitrogen, *Cr *creatinine, *UA *uric acid, *Aβ*_*1–42*_ amyloid-β1–42, *Aβ*_*1–40*_ amyloid-β1–40, *T-tau* total tau, *P-tau* phosphorylated tau

Aging is a significant risk factor for AD; thus, we wondered whether B2M levels reflect typical aging. B2M levels were found to rise with age, as shown by a statistically significant positive association (*β* = 0.004, *P* < 0.001). (Additional file 1: Fig. S2). The findings confirmed that B2M varied substantially among the different age categories (elderly: 2.04 ± 0.77 mg/l, *n* = 424; *P* = 0.005; middle age: 1.74 ± 0.69 mg/l, *n* = 422). (Additional file 1: Fig. S3, A). The B2M levels were not affected by sex (Additional file 1: Fig. S3, B) or *APOE* carrier status (all *P* > 0.05) (Additional file 1: Fig. S3, C).

### Differences in plasma B2M levels in relation to the various biomarker classifications

The variations in B2M levels between the four stages were compared using a biomarker classification framework, particularly for stage 0 (*n* = 315), stage 1 (*n* = 189), stage 2 (*n* = 92), and SNAP (*n* = 250), to identify the relationship between B2M changes and downstream processes of Aβ deposition and amyloid cascade (i.e., tau pathology and neurodegeneration).

**Fig. 1:**
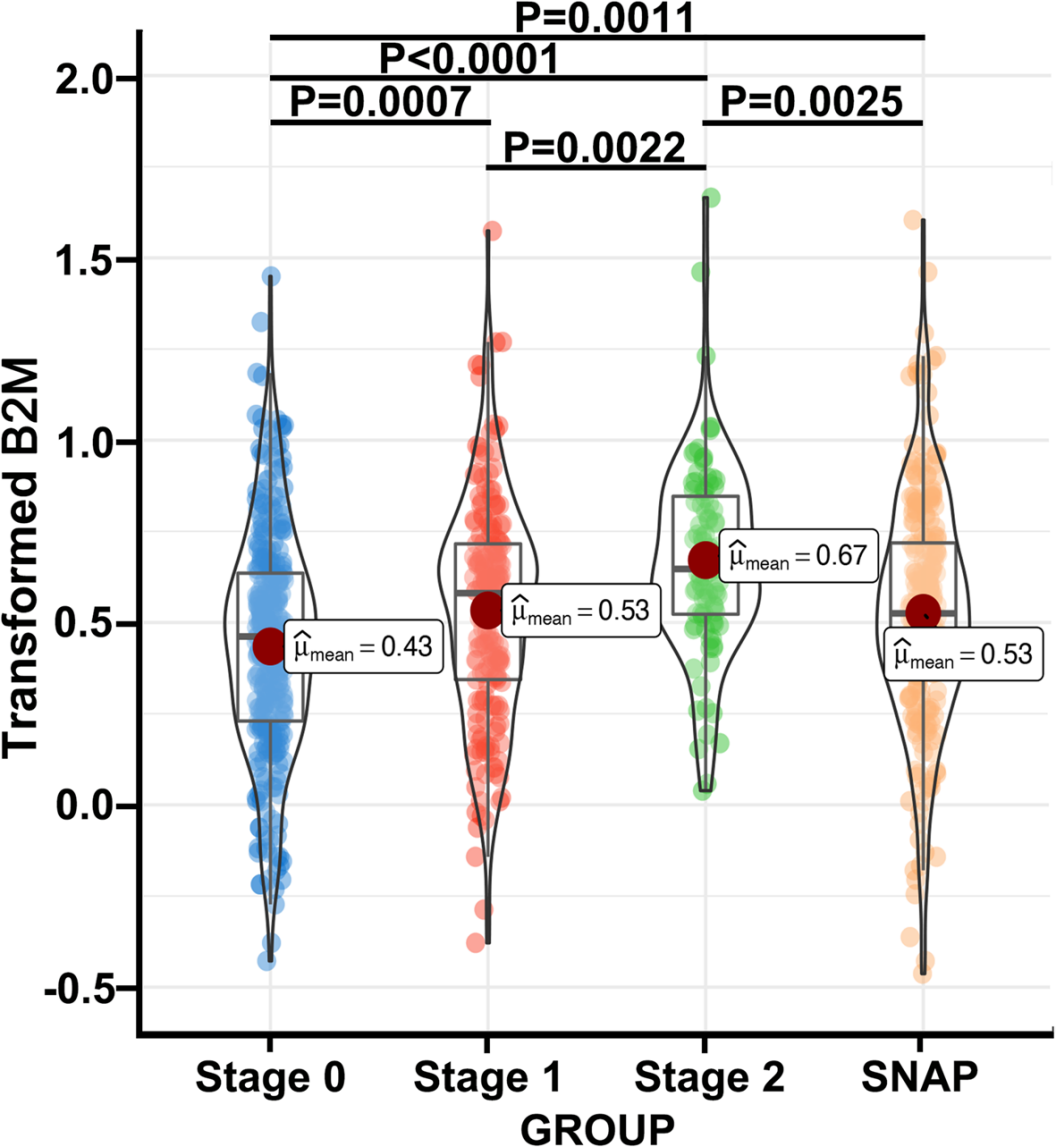
Plasma B2M in the biomarker classification. The cutoff values to define abnormal CSF AD biomarkers were < 205.82 pg/ml for Aβ_1–42_ (A +), > 48.73 pg/ml for P-tau (T +), or > 221.84 pg/ml for T-tau (T +). One-way ANOVA followed by Bonferroni post hoc analyses with adjustment for age was applied to examine the differences in plasma B2M across biomarker profiles in a biomarker framework. *Abbreviations*: B2M, β_2_-microglobulin; SNAP, suspected non-AD pathology

Significant differences in B2M levels were noted between the four groups according to the one-way ANCOVA results. Equally important, compared with the others, B2M was found to be significantly at its lowest level in the stage 0 group (Fig. 1). The B2M concentrations were significantly higher in stage 1 (*P* = 0.0007), stage 2 (*P* < 0.0001), and SNAP (*P* = 0.0011) groups than in stage 0 (Fig. 1). An ANCOVA model adjusting age was used since the study’s findings specified that age is a crucial factor impacting plasma B2M, and we acquired the same outcomes as before. Compared with the other groups, B2M was considerably lower in stage 0 (*P* < 0.001).

Differences in B2M levels between multiple groups were noted, with an increase in SNAPs compared with stage 0 (*P* = 0.0011). Then, B2M levels in SNAPs were found to have significantly decreased as opposed to stage 2 (*P* = 0.0025). These outcomes suggest elevated B2M levels are a superimposed response to amyloid and tau pathology or neuronal damage (as measured by CSF P-tau or T-tau levels).

### Relationship between plasma B2M with cognition and CSF AD biomarkers

**Fig. 2:**
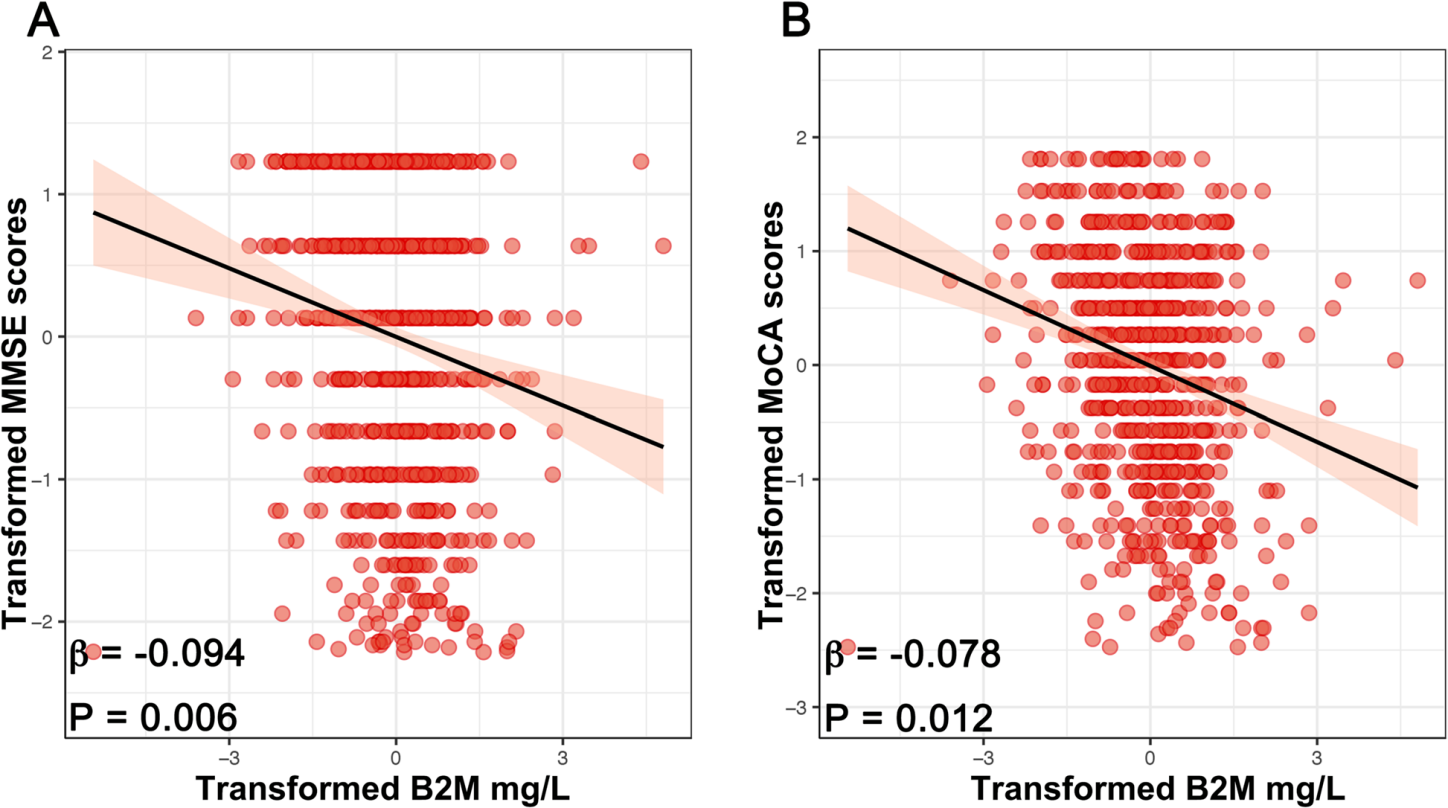
Associations of B2M and cognitive performance. Multiple linear regression models were used to examine the associations between the plasma B2M with MMSE (**A**) and MoCA (**B**), adjusting for age, sex, education level, and *APOE ɛ4* carrier statuses. *Abbreviations*: B2M, β_2_-microglobulin; MMSE, Mini-Mental State Examination; MoCA, Montreal Cognitive Assessment; *APOE*, *apolipoprotein E*

**Fig. 3:**
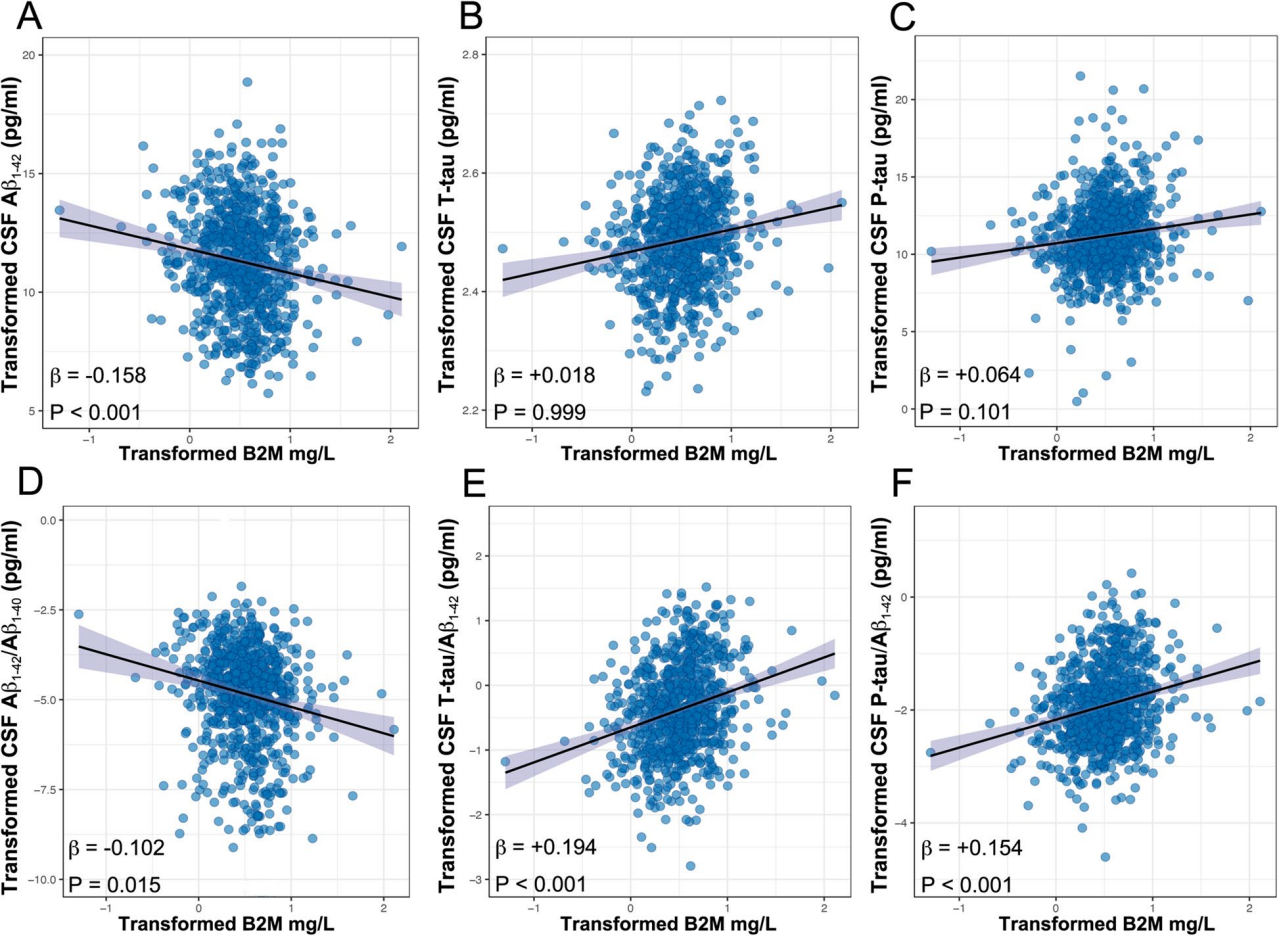
Associations between B2M and CSF AD biomarkers. Multiple linear regression models were used to examine the associations between the plasma B2M with cerebrospinal fluid (CSF) Aβ_1–42_ (**A**), T-tau (**B**), P-tau (**C**), Aβ_1–42_/Aβ_1–40_ (**D**) T-tau/Aβ_1–42_ (**E**), and P-tau/Aβ_1–42_ (**F**), adjusting for age, sex, education level, *APOE ɛ4* carrier statuses, and MMSE. *Abbreviations*: B2M, β_2_-microglobulin; *APOE*, *apolipoprotein E*

We used a linear regression model to examine the relationship between plasma B2M and cognition, adjusting for age, sex, education level, and *APOE ε4* carrier status. Increased plasma B2M was linked to lower levels of MMSE (*β* =  − 0.094, *P* = 0.006) and MoCA (*β* =  − 0.078, *P* = 0.012) in the entire sample of patients (Fig. 2). Next, the link between B2M and the CSF core biomarkers of AD was evaluated using a linear regression model, adjusting for age, sex, education level, *APOE ε4* status, and the MMSE score based on the dynamics of plasma B2M. Increased plasma B2M was correlated with lower levels of Aβ_1–42_ in the total sample of participants (*n* = 846) (*β* =  − 0.158, *P* < 0.001), whereas T-tau (*β* = 0.064, *P* = 0.101) and P-tau (*β* = 0.018, *P* = 0.999) were not associated (Fig. 3). Afterward, we surveyed the ratios of CSF amyloid and tau biomarkers and discovered that an increase in plasma B2M was correlated with both (a) a drop in CSF Aβ_1–42_/Aβ_1–40_ (*β* =  − 0.102, *P* = 0.015) and (b) an increase in T-tau/Aβ_1–42_ (*β* = 0.194, *P* < 0.001) and P-tau/Aβ_1–42_ (*β* = 0.154, *P* < 0.001) (Fig. 3). Upon executing additional correction factor adjustments, the sensitivity analysis exhibited that the aforementioned results remained consistent (Additional file 1: Table S1). These findings imply an early B2M response to the initial signs of neurodegeneration, with increased plasma B2M levels related to Aβ deposition independent of tau pathology and neurodegeneration.

### Interactions and stratified analyses by *APOE ε4* status, age, sex, CVF, and SCD

**Fig. 4:**
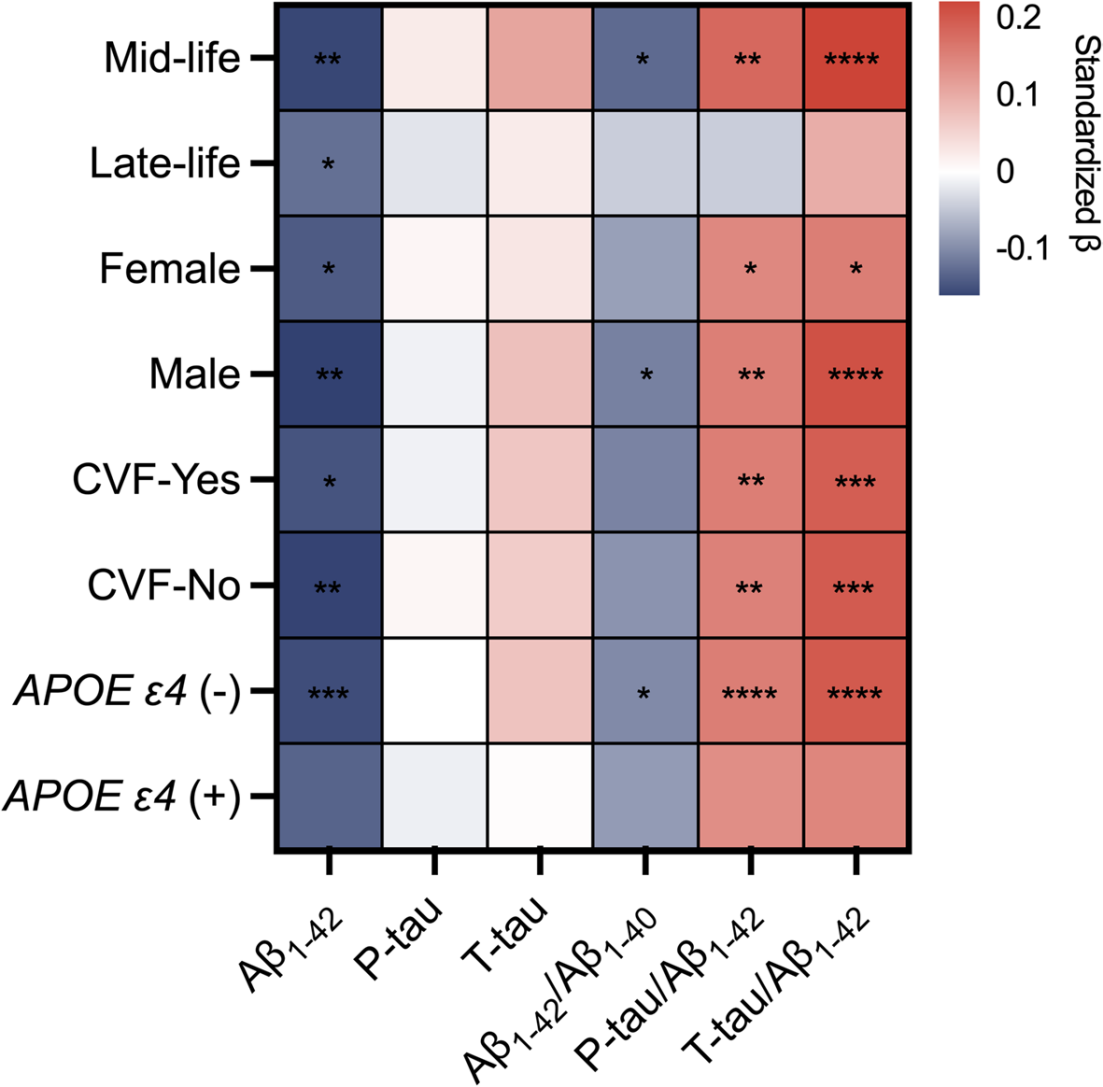
Heatmap for subgroup analyses of the association between B2M and CSF AD biomarkers. Multiple linear regression models were employed with adjustment for age, sex, years of education, *APOE ε4* status, and MMSE. In stratified analyses, stronger correlations were observed between B2M and biomarkers in middle-aged individuals and individuals without the *APOE ε4* allele. *Abbreviations*: CVF, cardiovascular factors; *APOE*, *apolipoprotein E*. *P < 0.05; **P < 0.01; ***P < 0.001; ****P < 0.0001

The interaction analysis expressed that the association between B2M and CSF AD biomarkers was not affected by sex, age, years of education, CVF, SCD, and *APOE ε4* status (Additional file 1: Table S2). We performed additional stratified analyses and discovered that B2M was substantially connected with Aβ_1–42_ (*β* =  − 0.159, *P* = 0.005), Aβ_1–42_/Aβ_1–40_ (*β* =  − 0.129, *P* = 0.027), P-tau/Aβ_1–42_ (*β* = 0.181, *P* = 0.001), and T-tau/Aβ_1–42_ (*β* = 0.226, *P* < 0.001) in middle age. Meanwhile, only Aβ_1–42_ (*β* =  − 0.124, *P* = 0.03) was correlated with B2M in old age (Fig. 4). Then, B2M was strongly correlated with Aβ_1–42_ (*β* =  − 0.153, *P* < 0.001), Aβ_1–42_/Aβ_1–40_ (*β* =  − 0.101, *P* = 0.018), P-tau/ Aβ_1–42_ (*β* = 0.155, *P* < 0.001), and T-tau/ Aβ_1–42_ (*β* = 0.199, *P* < 0.001) biomarkers in non-*APOE ε4* status (Fig. 4). However, B2M in *APOE ε4* status did not correlate with the biomarkers. The results did not differ in the sex and CVF subgroups (Fig. 4).

### Causal mediation analyses

**Fig. 5:**
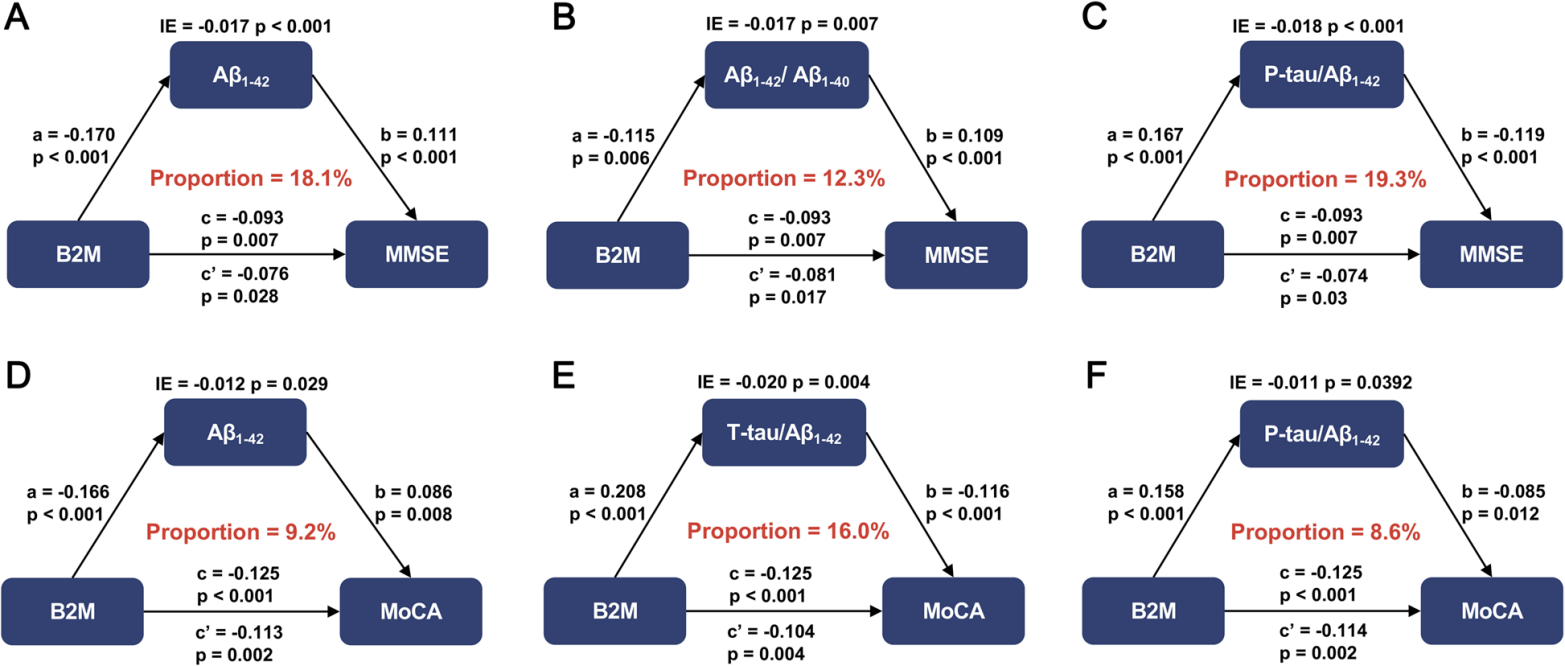
Mediation analyses of AD biomarkers between B2M and cognition. Mediation analyses with 10,000 bootstrapped iterations were used to examine the mediation effects of Aβ pathologies on cognition. Aβ_1–42_, Aβ_1–42_/Aβ_1–40_, and P-tau/Aβ_1–42_ mediated the relationship between B2M and MMSE (**A**–**C**). Aβ_1–42_, P-tau/Aβ_1–42_, and T-tau/Aβ_1–42_ mediated the relationship between B2M and MoCA (**D**–**F**). Each model path was adjusted for age, sex, years of education, *APOE ε4* status, and MMSE. *a* is the effect of the independent variable on mediators; *b* is the effect of mediators on dependent variables after controlling the influence of independent variables; *c* is the total effect of independent variables on dependent variables; *c*′ is the direct effect. *Abbreviations*: B2M, β_2_-microglobulin; P-tau, phosphorylated tau protein; T-tau, total tau protein; Aβ, amyloid β; *APOE*, *apolipoprotein E*

The direct, indirect, and total effects of Aβ_1–42_ on cognition were all statistically significant (*P* < 0.05), highlighting that the connection of B2M with MMSE and MoCA was solely mediated by Aβ_1–42_, with a mediating ratio of 18.1% and 9.2%, respectively (Fig. 5). The Aβ_1–42_/Aβ_1–40_ (mediating ratio = 12.3%) and P-tau/Aβ_1–42_ (mediating ratio = 19.3%) mediated the relationship of B2M with MMSE, while P-tau/Aβ_1–42_ (mediating ratio = 8.6%) and T-tau/Aβ_1–42_ (mediating ratio = 16.0%) mediated the correlation of B2M with MoCA (Fig. 5).

## Discussion

In this study, we aimed to assess the alterations in plasma B2M levels during the early stages of AD by incorporating a biological marker-based classification scheme. Using this classification system, we observed dynamic changes in B2M levels that progressively increased with pathological evolution. Furthermore, our study extended previous findings by exploring the relationship between B2M and the cerebrospinal fluid (CSF) AD biomarkers and cognition, which was not previously reported in the literature. Our results showed that although neurodegeneration or tau pathology was not related to B2M, the increase in B2M was related to the pathological changes of Aβ (low CSF Aβ_1–42_). Given the association between cognitive decline and elevated B2M levels, as well as the role of Aβ pathology in mediating the impact of B2M on cognition, the potential role of B2M in the pathogenesis of AD appears to be substantiated.

To evaluate the alterations in factor B2M related to cognitive functions and aging [13], we combined clinical analysis and biomarker-based categorization in this study. More particularly, using this categorization method enabled us to inspect the connection between pro-aging variables and the pathophysiology of AD, including Aβ_1–42_, tau pathology, and neurodegeneration. Our findings contribute to the existing literature by demonstrating that B2M levels dynamically change in response to certain pathogenic events. B2M was shown to be correlated with both an increase in Aβ pathology (defined as low CSF Aβ_1–42_) and a decline in cognitive scores (MMSE and MoCA). B2M, however, had no connection to tau pathology or neurodegeneration. Having said that, our results seem to support the theory that B2M is a pro-aging factor [33]. The accumulation of B2M in the blood promotes age-related cognitive dysfunction [13], signifying that B2M may be a therapeutic target for AD.

Our study details that even in the absence of tau pathology, higher B2M concentrations are associated with abnormal CSF Aβ_1–42_ levels, as indicated by the difference in B2M between stages 0 and 1. This implies that an increased B2M may induce the deposition of Aβ_1–42_, thereby leading to cognitive decline, as confirmed by our mediation analysis. In the absence of tau pathology, increased B2M appears to be a cause rather than a consequence of Aβ deposition, which is aligned with the findings of previous reports [8, 9, 34–38]. Additionally, considerable attention must be given to the most heterogeneous category, the SNAP group, given its potential to present symptoms that are not related to AD or neurodegeneration. In light of this, further research on this topic must extensively investigate the variations in B2M in neurodegenerative diseases apart from AD.

Exploring the relationship between plasma B2M and CSF AD biomarkers may generate a better overview of the pathogenicity by revealing concurrent processes in the brain. In the normal cognitive population, plasma B2M levels were negatively correlated with the CSF AD biomarkers Aβ_1–42_, but the correlation with T-tau and P-tau disappeared. These findings propose that alterations in plasma B2M may be associated with amyloid deposition and put forward that a facilitative response to amyloid deposition could be due to the increase in B2M during aging. Through subgroup analysis of individuals with or without *APOE ε4* status, B2M was found to be significantly associated with CSF AD biomarkers in *APOE ε4* carriers but not in non-carriers, suggesting a potential role for B2M in predicting future AD-related risks, particularly in individuals with non-*APOE ε4* status. Considering that B2M is a renal function indicator, previous studies have found that the *APOE ε4* carrier status does not affect the decline in renal function as a marker for individual dementia risk [39]. However, previous animal experiments have found that knocking out *APOE ε4* in mice leads to a decrease in peripheral clearance of Aβ_1–42_ [40], which may to some extent affect the relationship between B2M and Aβ_142_. Nevertheless, in our study, the number of *APOE ε4* carriers was limited to 110 (13.00%), which could lead to false negatives. Direct clinical evidence on the relationship between B2M and *APOE ε4* carrier status is still lacking, and further studies with adequate sample sizes and sufficiently long follow-up periods are required.

The mechanisms underlying the increase in plasma B2M early in Aβ pathology were unclear. One explanation is that B2M fibrils can release oligomers early on and rapidly spread to the cell membranes of the neighboring cells, triggering continuous events that result in sustained neuronal and glial cell damage [41]. Another justification is that B2M acts as a pro-senescence factor [33], which may partially activate amyloidosis, hence the high levels of B2M in stage 1. Presenting intracellular antigens to CD8 + T cells (cytotoxic T cells) necessitates all cells (excluding erythrocytes) to use B2M, which is part of the MHC I complex. The expression of MHCI-like molecules increases after aging, and the high expression of MHCI-like molecules impairs neuronal plasticity, neurite growth, and neurite regeneration [36, 42–48]. The results underline that impaired neurogenesis and cognitive dysfunction, which are age-related, are stimulated by B2M’s systemic accumulation in aging blood. On top of that, compared with the young mice, old mice experienced elevated B2M levels in their hippocampus and plasma [13], as substantiated by our subgroup analysis on age. Known as a likely pro-aging factor [33], B2M has been discovered to contribute to age-related cognitive impairment and aging progression (a previously unrecognized function) [13]. Our study reveals that B2M may be a risk factor for the early progression of AD, which is consistent with the existing literature [14, 15]. Nevertheless, despite the trend of elevated B2M in stage 2, we noted that B2M did not correlate precisely with tau pathology and neurodegeneration, which could be due to tau pathology being driven by the Aβ deposition, and the highest concentration of B2M in stage 2 was a consequence of the action of the Aβ deposition.

Numerous factors enhance the reliability of our study. First, this investigation examined the relationship between B2M and the CSF AD biomarkers and is the largest to date. Second, we utilized the AD diagnostic criteria from the NIA-AA study to classify AD biomarkers, ensuring the high quality of the study. Additionally, the CSF data were subjected to blinded quality control, and the results allowed statistical control for various possible confounders. However, the current study has several limitations that should be considered. Firstly, this was a cross-sectional study, which means that it cannot establish causality. Secondly, the results should be verified through larger longitudinal studies. Third, considering the fact that brain imaging data (e.g., PiB-PET or Tau-PET) may better reflect the association between AD pathology and B2M, future prospective neuroimaging cohorts would facilitate validating or supplementing evidence about the role of factor B2M. Lastly, the correlation between plasma B2M and cerebrospinal fluid AD-related biomarkers in the preclinical phase of AD remains to be further explored. Despite these limitations, the protective association found in this study provides valuable insights for future research into the potential causes and mechanisms of AD. Further examination of the relationship between plasma B2M and AD biomarkers may help to clarify the role of B2M in AD.

## Conclusions

Overall, the study identified an association between plasma B2M and CSF AD biomarkers and a possible important role of Aβ pathology in the association between B2M and cognitive impairment. This information is crucial to reveal the pathological mechanisms of B2M involvement in dementia or AD. The connection between B2M and AD pathogenesis will be further strengthened by additional research to determine its underlying processes, which might lead to the identification of novel pathogenetic pathways and treatment targets.

## Supplementary Information

**Additional file 1:** **Fig. S1.** The Quantile-Quantile plot of B2M. **Fig. S2.** Associations of age and B2M. **Fig. S3.** Levels of B2M in the CSF biomarker classifications. **Table S1.** The linear relationships between β_2_-microglobulin and CSF biomakers. **Table S2.** Interaction analysis of β_2_-microglobulin with multiple factors.

## Data Availability

The datasets used and analyzed in the current study are available from the corresponding authors upon reasonable request.

## Abbreviations

B2M: β_2_-Microglobulin
CSF: Cerebrospinal fluid
AD: Alzheimer’s disease
CABLE: Chinese Alzheimer’s Biomarker and LifestylE study
*APOE** ε4*: Apolipoprotein E genotype ε4
Aβ: Amyloid β
P-tau: Phosphorylated tau
T-tau: Total tau
MMSE: Mini-Mental State Examination
MoCA: Montreal Cognitive Assessment
CVF: Cardiovascular factors
