# Supplementary material for: Plasma β2-microglobulin and cerebrospinal fluid biomarkers of Alzheimer’s disease pathology in cognitively intact older adults: the CABLE study
Source: Alzheimers Res Ther. 2023 Apr 1;15:69. doi: 10.1186/s13195-023-01217-6 (PMC10067214; doi:10.1186/s13195-023-01217-6)
Supplement: Supplementary file 1 — Additional file 1: Fig. S1. The Quantile-Quantile plot of B2M. Fig. S2. Associations of age and B2M. Fig. S3. Levels of B2M in the CSF biomarker classifications. Table S1. The linear relationships between β2-microglobulin and CSF biomakers. Table S2. Interaction analysis of β2-microglobulin with multiple factors. [file 13195_2023_1217_MOESM1_ESM.docx]

**Content:**

[Supplementary Figure 1. 1](#_Toc127802353)

[Supplementary Figure 2. 2](#_Toc127802354)

[Supplementary Figure 3. 4](#_Toc127802355)

[Supplementary Table 1. 5](#_Toc127802356)

[Supplementary Table 2. 6](#_Toc127802357)

Supplementary Figure 1. **The Quantile-Quantile plot of B2M.**


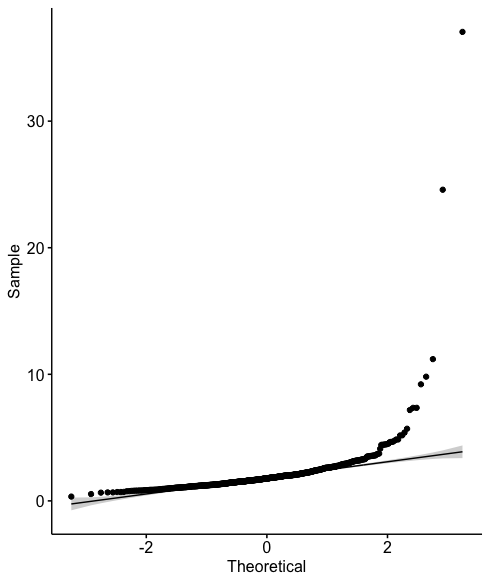


Sample quantiles were plotted against those theoretical quantiles under the null hypothesis (x-axis). QQ graph indicates that the data of B2M does not conform to the normal distribution.

Abbreviations: B2M, β_2_-microglobulin.

Supplementary Figure 2. **Associations of age and B2M.**


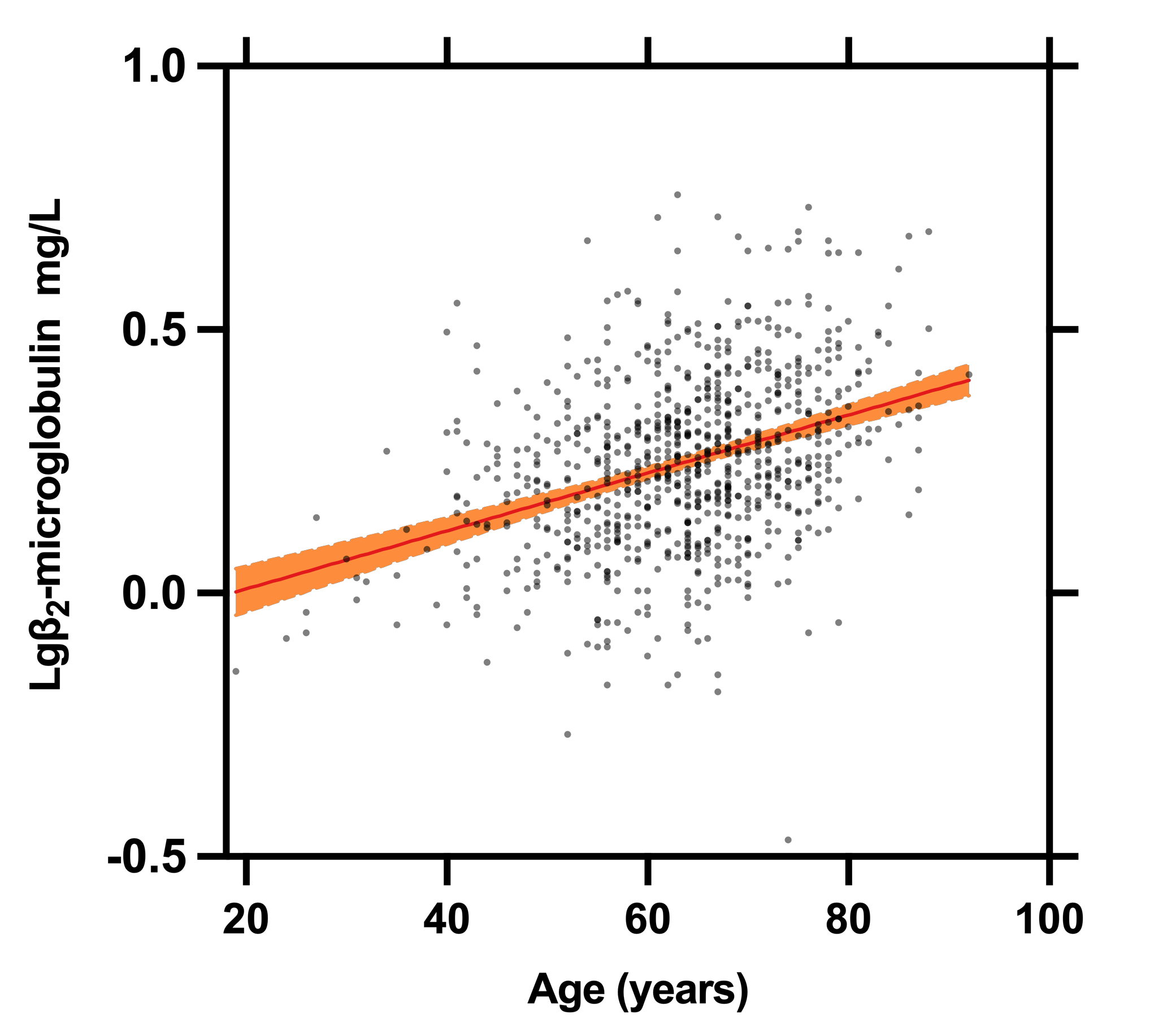


B2M levels are positively correlated with age.

Abbreviations: B2M, β_2_-microglobulin.

Supplementary Figure 3. **Levels of B2M in the CSF biomarker classifications.**

**B2M levels are associated with age (A) and are independent of gender (B) and *APOE* carrier status (C). P-values were assessed by student’s t test.**


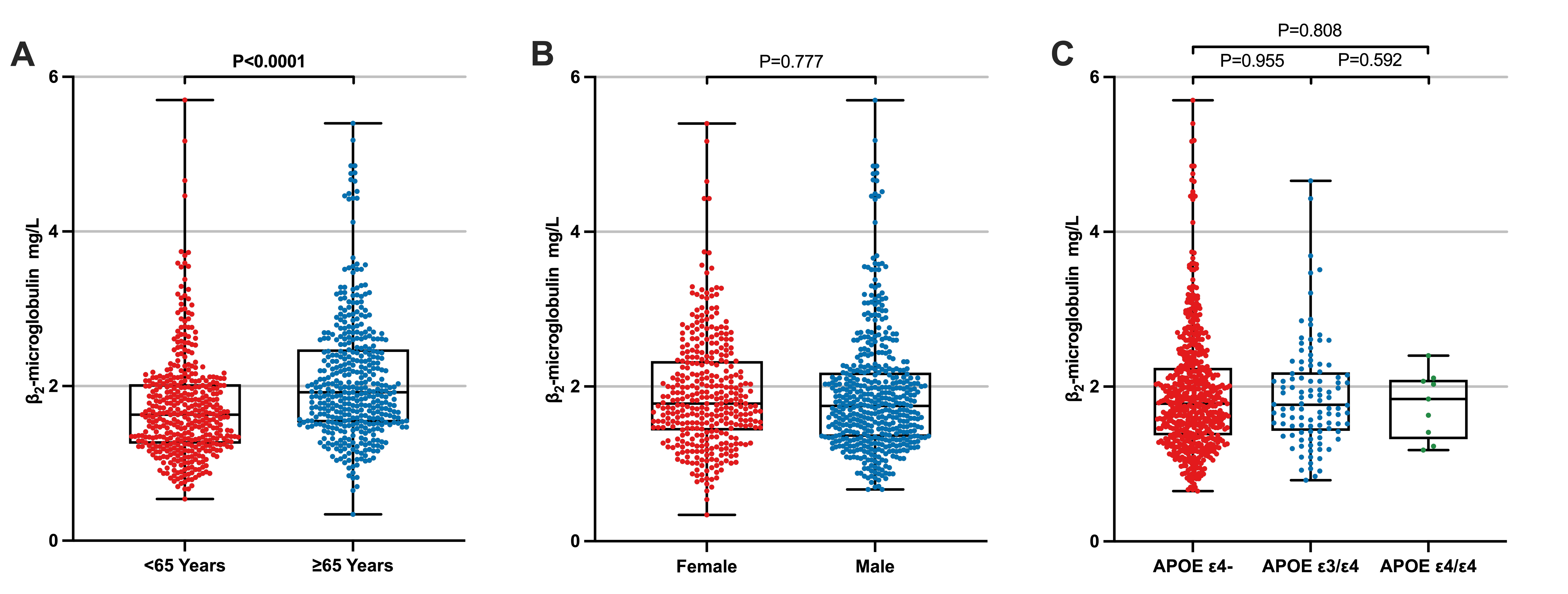


Abbreviations: B2M, β_2_-microglobulin; *APOE*, apolipoprotein E.

Supplementary Table 1. **The linear relationships between β_2_-microglobulin and CSF biomakers**

| The linear relationships between β_2_-microglobulin and CSF biomakers | | | | | | | | | |  |
| --- | --- | --- | --- | --- | --- | --- | --- | --- | --- | --- |
| CSF biomarkers |  | Model1 | |  | Model2 | |  | Model3 | |  |
|  |  | β | p |  | β | p |  | β | p |  |
| Aβ_1-42_ (pg/ml) |  | -1.101 | **<0.001** |  | -1.064 | **<0.001** |  | -1.301 | **<0.001** |  |
| T-tau (pg/ml) |  | 0.018 | 0.946 |  | -0.020 | 0.944 |  | 0.053 | 0.869 |  |
| P-tau (pg/ml) |  | 0.016 | 0.086 |  | 0.013 | 0.160 |  | 0.012 | 0.286 |  |
| Aβ_1-42_/Aβ_1-40_ |  | -0.574 | **0.005** |  | -0.509 | **0.015** |  | -0.632 | **0.009** |  |
| T-tau/Aβ_1-42_ |  | 0.363 | **<0.001** |  | 0.349 | **<0.001** |  | 0.440 | **<0.001** |  |
| P-tau/Aβ_1-42_ |  | 0.444 | **<0.001** |  | 0.417 | **<0.001** |  | 0.476 | **<0.001** |  |
| Model 1: adjusted for age, gender, education year, *APOE ε4* status.  Model2: model 1+ MMSE, alcohol consumption, smoking, diabetes mellitus, hypertension, hyperlipidemia, history of stroke, history of cardiovascular disease.  Model 3: model 2+ serum uric acid, blood urea nitrogen, blood creatinine, blood urea nitrogen/blood creatinine. APOE, apolipoprotein E; Aβ, amyloid-β; MMSE, China modified Mini-Mental State Examination; CSF, cerebrospinal fluid; P-Tau, phosphorylated tau; T-Tau, total tau. | | | | | | | | | |  |
|  |  |  |  |  |  |  |  |  |  |  |
|  |  |  |  |  |  |  |  |  |  |  |
|  |  |  |  |  |  |  |  |  |  |  |
|  |  |  |  |  |  |  |  |  |  |  |

Supplementary Table 2. **Interaction analysis of β_2_-MG with multiple factors.**

| Interaction analysis of β_2_-MG with multiple factors. | | | | | | | | |  |
| --- | --- | --- | --- | --- | --- | --- | --- | --- | --- |
|  |  | Aβ_1-42_ | Aβ_1-40_ | P-tau | T-tau | Aβ_1-42_/Aβ_1-40_ | P-tau/Aβ_1-42_ | T-tau/Aβ_1-42_ |  |
| Age | β | 0.0051 | -0.0002 | 0.0060 | 0.0012 | 0.0072 | -0.0036 | -0.0047 |  |
|  | P | 0.6208 | 0.9831 | 0.5372 | 0.9026 | 0.4972 | 0.7158 | 0.6390 |  |
| Sex | β | -0.0912 | 0.0107 | -0.0537 | 0.0832 | -0.0002 | 0.0810 | 0.1786 |  |
|  | P | 0.7088 | 0.9656 | 0.8142 | 0.7199 | 0.9995 | 0.7290 | 0.4471 |  |
| *APOE* | β | -0.0152 | 0.3097 | 0.2915 | 0.0564 | -0.3155 | 0.1634 | 0.0615 |  |
|  | P | 0.9663 | 0.3950 | 0.3852 | 0.8690 | 0.3883 | 0.6345 | 0.8588 |  |
| Edu | β | 0.0070 | -0.0307 | -0.0121 | 0.0187 | 0.0252 | -0.0077 | 0.0055 |  |
|  | P | 0.7988 | 0.2727 | 0.6397 | 0.4757 | 0.3696 | 0.7714 | 0.8360 |  |
| CVF | β | -0.0333 | 0.0636 | 0.1601 | 0.0519 | -0.0059 | 0.0739 | 0.0771 |  |
|  | P | 0.8904 | 0.7956 | 0.4793 | 0.8214 | 0.9808 | 0.7493 | 0.7402 |  |
| SCD | β | 0.0076 | -0.1668 | 0.3156 | 0.2844 | 0.2394 | 0.1784 | 0.1672 |  |
|  | P | 0.9752 | 0.5012 | 0.1672 | 0.2195 | 0.3341 | 0.4443 | 0.4748 |  |
| Abbreviations: β2-MG, β_2_-microglobulin; *APOE*, apolipoprotein E; Aβ_1-42_, amyloid-β_1–42_; Aβ_1-40_, amyloid-β_1–40_; T-tau, total Tau; P-tau, phosphorylated Tau; CVF, cardiovascular factors (including stroke, coronary heart disease, high blood pressure, diabetes); SCD, subjective cognitive decline. | | | | | | | | |  |
|  |  |  |  |  |  |  |  |  |  |
|  |  |  |  |  |  |  |  |  |  |
